# Botulinum Neurotoxin F Subtypes Cleaving the VAMP-2 Q^58^–K^59^ Peptide Bond Exhibit Unique Catalytic Properties and Substrate Specificities

**DOI:** 10.3390/toxins10080311

**Published:** 2018-08-01

**Authors:** Stefan Sikorra, Martin Skiba, Martin B. Dorner, Jasmin Weisemann, Mirjam Weil, Sylvia Valdezate, Bazbek Davletov, Andreas Rummel, Brigitte G. Dorner, Thomas Binz

**Affiliations:** 1 Institute of Cell Biochemistry, OE 4310, Hannover Medical School, Carl-Neuberg-Straße 1, 30625 Hannover, Germany; S.Sikorra@kabelmail.de; 2 Biological Toxins (ZBS 3), Centre for Biological Threats and Special Pathogens, Robert Koch Institute, 13353 Berlin, Germany; SkibaM@rki.de (M.S.); DornerM@rki.de (M.B.D.); miri.weil@gmx.de (M.W.); 3 Institute of Toxicology, OE 5340, Hannover Medical School, Carl-Neuberg-Straße 1, 30625 Hannover, Germany; Weisemann.Jasmin@mh-hannover.de (J.W.); Rummel.Andreas@mh-hannover.de (A.R.); 4 Reference and Research Laboratory for Taxonomy, Spanish National Centre of Microbiology, Institute of Health Carlos III, 28220 Madrid, Spain; svaldezate@isciii.es; 5 Department of Biomedical Science, University of Sheffield, Western Bank, Sheffield S10 2TN, UK; b.davletov@sheffield.ac.uk

**Keywords:** *Clostridium botulinum*, botulinum neurotoxin, serotype F, subtype, vesicle associated membrane protein 2 (VAMP-2), synaptobrevin, Zn^2+^ protease

## Abstract

In the recent past, about 40 botulinum neurotoxin (BoNT) subtypes belonging to serotypes A, B, E, and F pathogenic to humans were identified among hundreds of independent isolates. BoNTs are the etiological factors of botulism and represent potential bioweapons; however, they are also recognized pharmaceuticals for the efficient counteraction of hyperactive nerve terminals in a variety of human diseases. The detailed biochemical characterization of subtypes as the basis for development of suitable countermeasures and possible novel therapeutic applications is lagging behind the increase in new subtypes. Here, we report the primary structure of a ninth subtype of BoNT/F. Its amino-acid sequence diverges by at least 8.4% at the holotoxin and 13.4% at the enzymatic domain level from all other known BoNT/F subtypes. We found that BoNT/F9 shares the scissile Q^58^/K^59^ bond in its substrate vesicle associated membrane protein 2 with the prototype BoNT/F1. Comparative biochemical analyses of four BoNT/F enzymatic domains showed that the catalytic efficiencies decrease in the order F1 > F7 > F9 > F6, and vary by up to a factor of eight. K_M_ values increase in the order F1 > F9 > F6 ≈ F7, whereas k_cat_ decreases in the order F7 > F1 > F9 > F6. Comparative substrate scanning mutagenesis studies revealed a unique pattern of crucial substrate residues for each subtype. Based upon structural coordinates of F1 bound to an inhibitor polypeptide, the mutational analyses suggest different substrate interactions in the substrate binding channel of each subtype.

## 1. Introduction

Botulinum neurotoxins (BoNTs) are produced by the Gram-positive bacterium, *Clostridium botulinum*, and some other strains of *C. baratii* and *C. butyricum*. Historically, they are differentiated based on serum neutralization properties into seven accepted serotypes (A to G). Recently, a number of potentially novel serotypes emerged, namely BoNT/H (also known as BoNT/FA or BoNT/HA), BoNT/X, and BoNT/En (also known as eBoNT/J) [1,2,3]. Additionally, a still rapidly growing number of BoNT subtypes were identified in the last decade based on sequence diversity, and were assigned to serotypes A, B, E, and F [4,5]. Here, BoNT sequences with at least 2.6% difference in amino-acid sequence were designated as individual subtypes [6]. BoNT/F represents the serotype with the largest sequence variations among its subtypes. The eight characterized subtypes (BoNT/F1–F8; [7,8]) to date exhibit as far as 36% divergence (BoNT/F5, strain Mendoza-CDC54074 versus BoNT/F7, strain Oregon-CDC59837). Interestingly, the individual F subtypes are produced by three different bacteria of the genus *Clostridium*. Subtypes F1–F5 and F8 are produced by *C. botulinum* Group I which is almost indistinguishable from *C. sporogenes*, while F6 is produced by *C. botulinum* Group II which is related to *C. taeniosporum*, and F7 is the only BoNT made by *C. baratii* [9]. While the relevance of BoNT subtypes is still under investigation, it was demonstrated that subtypes of a given serotype might differ not only by sequence, but also by their biological activity, e.g., affinity to receptors or kinetics of substrate cleavage [10,11,12,13,14].

BoNTs are synthesized as single-chain proteins of 150 kDa and are subsequently processed to yield an N-terminal enzymatic light chain (LC) of ~50 kDa and a heavy chain of ~100 kDa. The heavy chain mediates highly specific binding to neuronal cells, and after endocytosis transfer of the enzymatic LC across the vesicular membrane. In the neuronal cytosol, the LCs act as zinc metalloproteases and generally hydrolyze soluble N-ethyl-maleimide sensitive factor attachment protein receptors (SNAREs), i.e., specific members of the vesicle associated membrane protein (VAMP; also designated synaptobrevin), synaptosomal-associated protein of 25 kDa (SNAP-25), or syntaxin families. Each serotype hydrolyzes a unique peptide bond of its substrate(s). All subtypes studied so far share the scissile substrate peptide bond with their BoNT prototype. The sole exception is LC/F5 which was demonstrated to cleave the bond L^54^–E^55^ of VAMP-2 [11], whereas all other analyzed F subtypes (F1, F2, F4, F6, and F7) cleave the Q^58^–K^59^ bond [15,16,17,18,19]. The peculiar cleavage activity of BoNT/F5 is explainable by the very high sequence diversity (about 53%) of LC/F5 from all other LC/Fs (Table S1). LC/F5 exhibits high sequence identity (approx. 80%) with the LC of the newly emerged BoNT/H (also known as BoNT/FA, BoNT/HA). Both LCs target the same peptide bond in VAMP-2 [11,20]. The rather low sequence identity of LC/F5 with all other LC/F subtypes (46–48%) and the high sequence identity of BoNT/F5 heavy chain with BoNT/F2 (89%) suggest that BoNT/F5 is a mosaic BoNT. For all BoNT sero- and subtypes, cleavage of any SNARE leads to inhibition of acetylcholine release, thereby causing flaccid paralysis that can lead to respiratory failure and death [5].

BoNTs are considered as the most toxic natural substances known and are categorized as tier 1 select agents as they represent potential bioweapons [21]. The 50% lethal doses for susceptible mammals are in the range of one nanogram per kg of body weight [22]. However, for 25 years, several BoNTs (firstly, BoNT/A, and more recently, also BoNT/B) served as effective pharmaceuticals for the treatment of medical conditions caused by hyperactivity of cholinergic nerve terminals. Initially approved for the treatment of blepharospasm, hemifacial spasm, and strabismus, the range of applications steadily expanded, and now also includes autonomic and other non-neuronal uses [23,24,25]. Additional serotypes are under investigation for potential use as pharmaceuticals, and new fields of application including non-neuronal systems are being explored. Moreover, it seems advisable to also analyze BoNT subtypes as they could outperform respective conventional BoNTs with respect to potency, onset, and duration of action, substrate selectivity, etc.

In the present study, we established the amino-acid sequence of a novel BoNT/F subtype (BoNT/F9), and performed a comparative analysis of the enzymatic activity of the prototypical LC/F1 and LC/F6, LC/F7, and LC/F9. We demonstrate that the three subtypes hydrolyze the Q^58^–K^59^ bond of VAMP-2 like LC/F1. However, LC/F7 and LC/F9 exhibit somewhat lower catalytic activities in hydrolyzing VAMP-2, but are nevertheless basically of interest for more detailed characterization of their properties.

## 2. Results

### 2.1. The Newly Identified BoNT Is Most Similar to Subtype BoNT/F3

**Figure 1:**
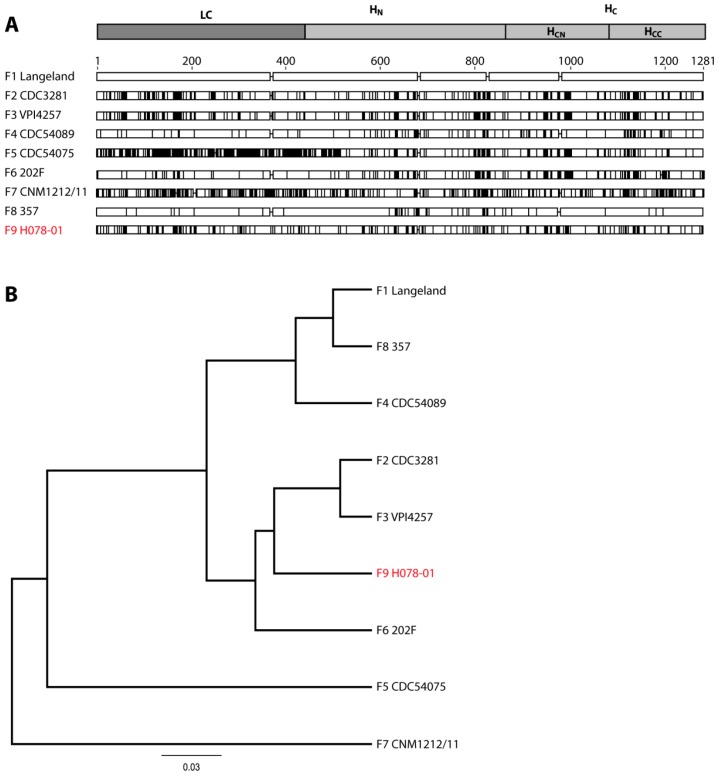
Comparison and relationship of botulinum neurotoxin (BoNT)/F subtypes. (**A**) Alignment of amino-acid substitutions of all known BoNT/F subtypes versus BoNT/F1. BoNT/F1 amino-acid sequence of strain Langeland as a prototype was compared to representatives of subtypes BoNT/F2 to F8 and the novel BoNT/F9. Sequence differences are indicated by vertical lines. The cartoon indicates the domain of the light chain (LC), the N-terminal part (H_N_), and the C-terminal part (H_C_) of the heavy chain (HC). The latter can be further subdivided into an N-terminal (H_CN_) and C-terminal (H_CC_) subdomain. (**B**) Dendrogram generated from the full-length amino-acid sequences representing different F subtypes, illustrating their locations within the serotype F. The distances illustrate the diversity within the serotype with the scale bar indicating the average substitution per site.

Routine screens of honey offered in German grocery stores for the presence of BoNT-producing clostridia were conducted in 2010. One preparation (origin European Union (EU) and non-EU countries) contained a *C. botulinum* strain designated H078-01. The nucleotide sequence and deduced amino-acid sequence of its neurotoxin gene was established. Comparison of the amino-acid sequence with other BoNT sequences available in GenBank revealed that its sequence is related to BoNT/F and exhibits between 91.6% and 69.0% identity to the published BoNT/F subtypes F1–F8 (Table 1) [7,8]. As differences at the amino-acid level exceeding 2.6% define new subtypes, the BoNT of strain H078-01 represents a novel subtype designated F9. Figure 1A depicts the differences in amino-acid sequences of all BoNT/F subtypes against the prototype F1 (strain Langeland [8]). The differences of BoNT/F9 versus BoNT/F1 are almost uniformly distributed among the toxin domains. The amino-acid sequence identities among BoNT/F9 and all known subtypes, including the mosaic BoNT/H (also known as BoNT/FA, BoNT/HA), are listed in Table 1. BoNT/F9 belongs to a cluster formed by F2, F3, and F6 (Figure 1B), and shows the highest identity to F3 and the lowest to F7 (a subtype expressed by *C. baratii*) for the holotoxin and subdomains alike. Interestingly, the subtypes in the cluster formed by F2, F3, F6, and F9 are produced by two different bacteria, *C. botulinum* Group I (F2, F3), which is almost indistinguishable from *C. sporogenes*, whereas F6 is made by *C. botulinum* Group II, which is more closely related to *C. taeniosporum*. Based on its 16S ribosomal DNA (rDNA) sequence, the strain producing the novel subtype F9 belongs to *C. botulinum* Group I.

### 2.2. The Novel BoNT/F9 LC Hydrolyzes the Q^58^–K^59^ Peptide Bond of VAMP-2

**Figure 2:**
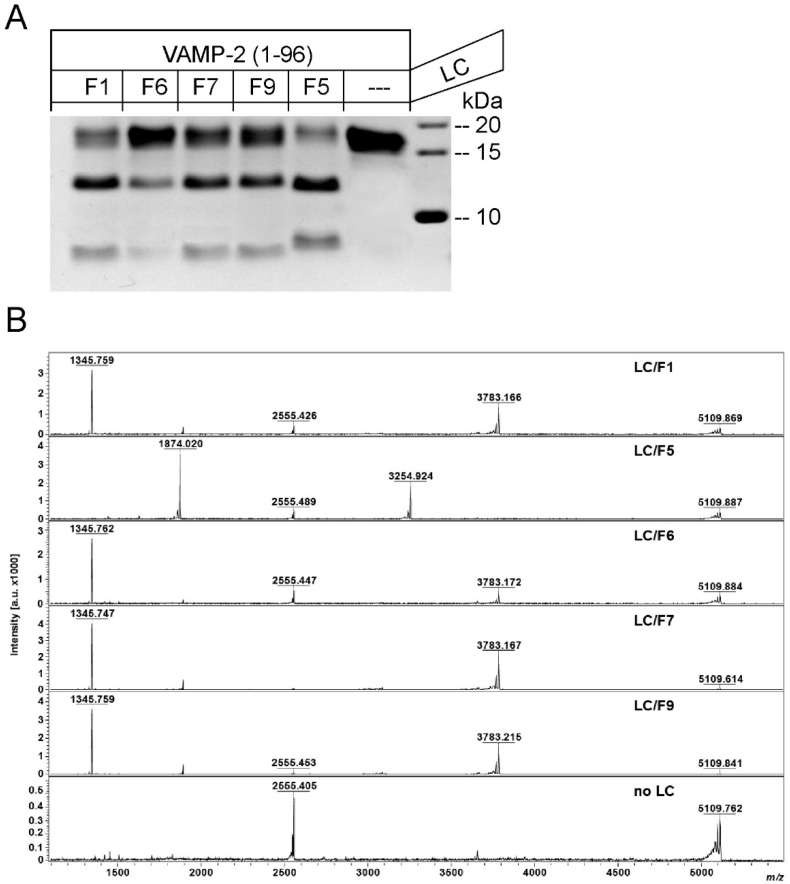
Cleavage of vesicle associated membrane protein 2 (VAMP-2) by light chain (LC)/F1, LC/F5, LC/F6, LC/F7, and LC/F9. (**A**) SDS-PAGE analysis of VAMP-2 cleaved by various BoNT/F LCs. VAMP-2 (amino acids 1–97) was incubated with LC/F of the indicated subtypes for 1 h at 37 °C in toxin assay buffer, and was subsequently subjected to SDS-PAGE and Coomassie staining. LC/F preparations were used at the following final concentrations: LC/F1, 0.5 nM; LC/F6, 3 nM; LC/F7, 0.5 nM; and LC/F9, 3 nM. These concentrations roughly correspond to LC doses that yield 60% cleavage of VAMP-2 at the given conditions. LC/F5 was included as control and applied at a final concentration of 100 nM. The C-terminal cleavage fragments migrated faster than the N-terminal cleavage fragments. The intact VAMP-2 variant used exhibits an apparent molecular weight of about 18 kDa. (**B**) Mass spectra for the reactions of LC/F1, LC/F5, LC/F6, LC/F7, and LC/F9 with VAMP-2 peptide T^27^SNRRLQQTQAQVDEVVDIMRVNVDKVLERDQKLSELDDRADAL^70^. Mass peaks at 1345.8 *m*/*z* and 3783.2 *m*/*z* indicate C- and N-terminal, respectively, cleavage products of the substrate generated by LC/F1, F6, F7, and F9, whereas mass peaks at 1874.0 *m*/*z* and 3254.9 *m*/*z* indicate cleavage by LC/F5. Intact VAMP-2 peptide shows a mass peak at 5109.8 *m*/*z* and 2555.4 *m*/*z* for the doubly charged ion.

The so far examined BoNT/F subtypes F1, F2, F4, F6, and F7 cleave VAMP-2 at the Q^58^–K^59^ peptide bond [15,16,17,18,19], whereas BoNT/F5 hydrolyzes VAMP-2 at L^54^–E^55^ [11]. LC/F9 exhibits considerable sequence divergence to other F subtypes. In addition, the scissile peptide bond of its closest relative BoNT/F3 was not determined. Therefore, we first investigated whether BoNT/F9 hydrolyzes VAMP-2 at a well-established peptide bond or at a novel position. To this end, the LC domains of F1 (*C. botulinum* Group I strain Langeland), F6 (*C. botulinum* Group II strain 202F), F7 (*C. baratii* strain CNM1212/11), and F9 (*C. botulinum* Group I strain H078-01) were cloned and expressed. Firstly, the BoNT/F substrate VAMP-2 was studied by in vitro cleavage assays, including F1, F5, F6, and F7. Cleavage fragments generated by LC/F9 showed virtually identical migration in SDS-PAGE compared to those of F1, F6, and F7, and were clearly distinct from cleavage fragments generated by LC/F5 (Figure 2A). Thus, F9 seemingly shares the scissile peptide bond with LC/F1. To exactly determine the cleavage position of F9, we next employed the Endopep-MS assay. To this end, recombinant LCs were incubated with a peptide substrate (amino acids 27–70) derived from VAMP-2 of 5109.6 Da (doubly charged peptide substrate peak occurs at *m*/*z* 2555.4; Figure 2B; [11]). The molecular masses of the C- and N-terminal cleavage products were then accurately identified by mass spectrometry (MS) allowing for deduction of the cleavage site. As expected, F1, F6, and F7 cleaved this peptide at the Q^58^–K^59^ bond, generating an N-terminal cleavage product of 3783.0 Da and a C-terminal product of 1345.7 Da. Cleavage by LC/F9 resulted in identical products, thus identifying Q^58^–K^59^ as the BoNT/F9 cleavage site, whereas F5 cleavage at L^54^–E^55^ yielded 3254.9 Da (N-terminal) and 1874.0 Da (C-terminal) fragments (Figure 2B).

### 2.3. BoNT/F Subtypes 1, 6, 7, and 9 Exhibit Significantly Different Enzymatic Properties

The cleavage process of VAMP-2 can be described as a sequence of consecutive steps leading eventually to optimal substrate positioning for peptide-bond hydrolysis. This involves different regions of LC, even those remote to the catalytic center. Thus, amino-acid changes in various LC regions may affect substrate turnover, and were shown to result in different substrate requirements and activities of LC/F subtypes; e.g., a peptide substrate (VAMP-2 amino acids 32–70) was readily cleaved by F1, F2, or F6, but not by F7, while full-length VAMP-2 or an N-terminally extended peptide (amino acids 27–70) were efficiently cleaved by all of them [15]. To elucidate whether the observed considerable sequence divergences resulted in different enzymatic properties, we determined the kinetic parameters in in vitro cleavage assays using the full cytoplasmic domain of VAMP-2. To rank the catalytic activity of LC/F9, we also determined those of LC/F6 and LC/F7 to which LC/F9 exhibits 80.6% and 60.4% sequence identity, respectively. LC/F1 served as reference. Table 2 shows that the K_M_ of LC/F9 is about 1.5-fold higher than K_M_ of LC/F1, but significantly lower compared to LC/F6 and LC/F7 (~1.6-fold and ~1.7-fold, respectively). The k_cat_ of LC/F9 is almost two- to three-fold lower than the k_cat_ of LC/F1 and LC/F7, but its turnover number exceeds that of LC/F6. Resulting from these data, the catalytic efficiency of LC/F9 ranks third, being 2.9-fold and 1.6-fold lower compared to LC/F1 and LC/F7, respectively, but 2.7-fold higher compared to LC/F6.

### 2.4. LC/F6, LC/F7, and LC/F9 Exhibit Unique Substrate Specificities

**Figure 3:**
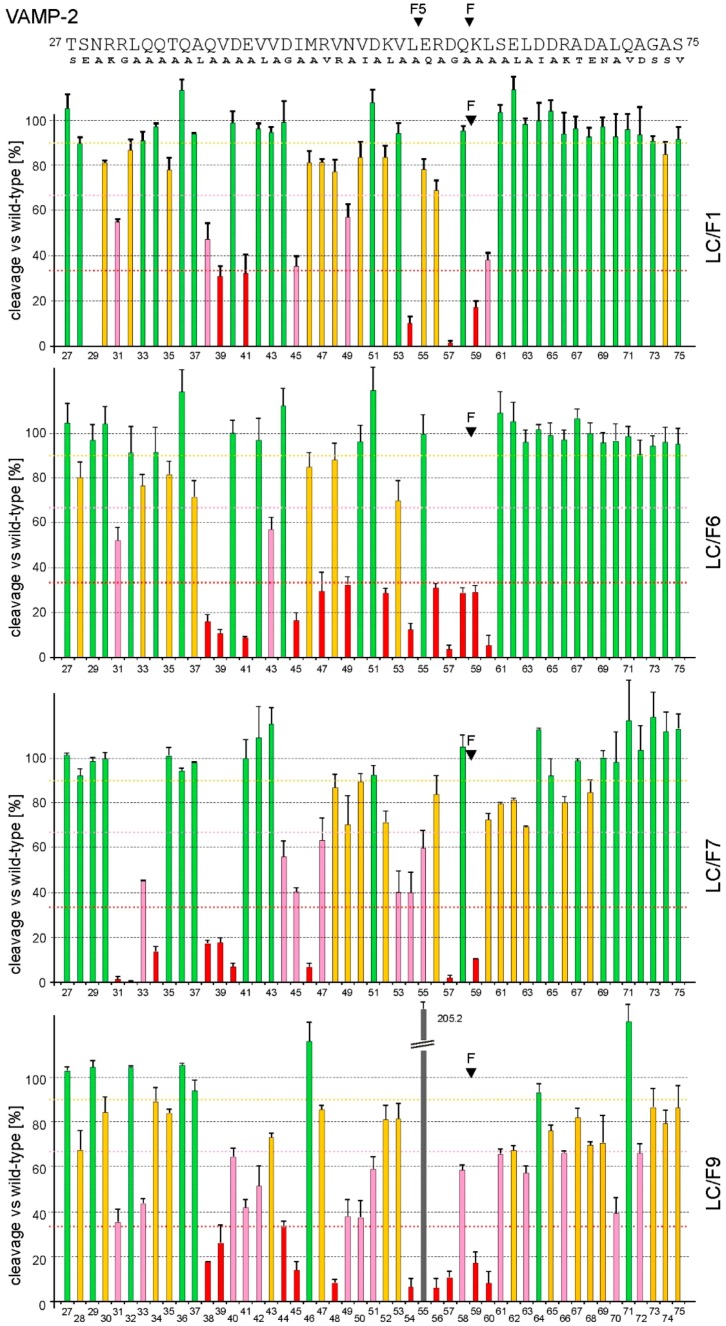
Cleavage analysis of VAMP-2 point mutants. Upper panel: The amino-acid sequence of the VAMP-2 region Thr-27 to Ser-75 is shown in single-letter code, and the peptide bonds hydrolyzed by BoNT/F1 and BoNT/F5 are marked “F” and “F5”, respectively. Mutations carried out in individual positions are depicted in smaller letters below. The majority of residues were converted to alanine. Lower panels: VAMP-2 mutants were radiolabeled by in vitro transcription/translation and incubated for 1 h at 37 °C in the presence of 0.2 nM LC/F1, 5 nM LC/F6, 0.5 nM LC/F7, or 2 nM LC/F9. Samples were analyzed by Tris/Tricine-PAGE using 15% gels. Columns represent percentages of cleavage versus the wild-type VAMP-2. Data represent means ± SD of three to ten independent experiments. Dotted lines in red, pink, and yellow specify thresholds of 10%, 33%, and 66% reduction in cleavability, respectively. The color code applied to the columns is as follows: green, no or less than 10% reduction in cleavability; yellow, more than 10% reduction in cleavability; pink, more than 33% reduction in cleavability; and red, more than 66% reduction in cleavability. The gray column displays a mutation causing significantly increased cleavability.

To establish the basis of these differences and to obtain information on the usefulness of LC/F subtypes as tools in basic research and possibly future clinical application, we next investigated the substrate specificity applying scanning mutagenesis of VAMP-2 in the region Thr-27 to Ser-75. This area was demonstrated to permit efficient cleavage by LC/F1 [19]. The majority of residues were converted to alanine. The mutants were generated as radiolabeled proteins, and cleavage was quantified subsequent to SDS-PAGE. The pattern of VAMP-2 amino acids whose mutation reduced substrate cleavage by LC/F6 was very similar to that of LC/F1 (Figure 3). Whereas ten mutations caused a reduction in cleavage by more than 33% in LC/F1, these ten plus five additional residues, Val-43, Arg-47, Lys-52, Arg-56, and Gln-58, diminished VAMP-2 cleavage in the case of LC/F6. The pattern of VAMP-2 residues involved in LC/F7 interaction is significantly different. Of the 16 positions whose mutation reduced cleavability, seven mutations, positions 31 to 34, 40, 44, and 46 caused much stronger effects on LC/F7 compared to LC/F1 and LC/F6. In contrast, mutation of Glu-41, Leu-54, and Leu-60 to alanine led to lesser effects. Efficient LC/F9 cleavage of VAMP-2 appears to be dependent on 24 substrate residues. Mutation of ten of them reduced cleavage by more than 66% (Figure 2, lowest panel, red columns). The pattern of substrate residues whose mutation affected VAMP-2 cleavage was again very different from that of LC/F1, LC/F6, and LC/F7. Mutation of eight of the 24 required amino acids led to much stronger reduction in the activity of LC/F9 compared to LC/F1. In contrast, exchanges of Met-46 by alanine yielded a much lesser effect, and Glu-55 to Gln even improved VAMP-2 cleavage by a factor of two. In summary, though the hydrolyzed peptide bond is the same, and the overall binding mode probably highly similar for LC/F1, LC/F6, LC/F7, and LC/F9, the set of VAMP-2 amino acids involved in the interaction is distinct for each of them.

To probe the substrate specificity, we studied cleavage of rat VAMP-1. Within the area required for interaction with LC/F, VAMP-1 exhibits two amino-acid changes versus rat VAMP-2: glutamic acid instead of Asp-40, and isoleucine instead of Met-46. LC/F1 cleaved VAMP-1 most efficiently, followed by LC/F7. At a final LC concentration of 3 nM, LC/F1 cleaved VAMP-1 completely, whereas incubation with LC/F7 caused about 50% conversion of the full-length VAMP-1 (Figure S1). This finding reflects the higher catalytic efficiency of LC/F1 in VAMP-2 cleavage, and additionally, the much higher sensitivity of LC/F7 to mutation at positions 40 and 46 (Figure 3). In contrast to proteolysis of VAMP-2, LC/F9 cleaved VAMP-1 only slightly more efficiently compared to LC/F6 (Figure S1). The reason may be that LC/F9 activity is, in contrast to LC/F6, sensitive to mutation at position 40.

## 3. Discussion

Coherent with the molecular characterization of BoNT serotypes, significant differences in amino-acid sequences between individual isolates within serotypes were recognized. Since modern sequencing technology became readily available, the number of characterized strains steeply increased, and their neurotoxins variants were consequentially classified into subtypes [30]. Here, we described the isolation of a *C. botulinum* strain obtained from honey. It expresses a novel BoNT/F subtype. Comparison of its amino-acid sequence with published sequences revealed closest similarity to BoNT/F3. As the holotoxin sequence differs by at least 8.4% from any known BoNT amino-acid sequence, it constitutes a novel subtype, to which the next consecutive number F9 was assigned.

For the majority of subtypes, it remains unclear to which extent the observed sequence variations affect their biological activity compared to the prototypical BoNT. Basically, any new subtype may possess features improving BoNTs with respect to their application as therapeutics or as tools for basic research. However, our knowledge on how sequence variations affect toxin binding, entry, catalytic activity, etc. is yet limited. Previous work on BoNT/A showed that subtypes exhibit unique properties with regards to receptor binding, enzymatic activity, overall toxicity, onset of toxicity, and duration of effects [10,12,13,14,31,32]. Subtypes of other serotypes are less well characterized. Regarding BoNT/F, some information on the catalytic activity of subtypes F5 and F7 exists [11,15,33,34]. Here, we studied the enzymatic properties of the LC of the new F9 subtype as a first step in its characterization, as well as that of F6. These properties turned out to be distinct among BoNT/F subtypes and are tentatively assigned below to differences in amino-acid sequences (differences in hydrophobicity and electrostatic potential; Figures S2 and S3).

### 3.1. Comparison of LC/F1 and LC/F6 Substrate Interactions

Prior crystallographic studies of LC/F1 bound to a VAMP-2-derived inhibitor peptide, comprising residues 22–57 plus a carboxyl-terminal D-cysteine residue, revealed possible interactions between LC/F and VAMP-2 [35]. Of the LC/F1 residues suggested to interact via their side chain with VAMP-2, two differ in LC/F6. Ile-149 is replaced by methionine and Ile-150 by leucine. The contribution of these residues to substrate binding (hydrophobic interactions) is yet to be investigated. However, it is unlikely that these changes alone explain the much higher K_M_ of LC/F6, since their suggested interaction partners in VAMP-2, Leu-32, and Gln-33 exhibit no essential role (Figure 3). Mutation of Leu-32 to alanine reduced cleavability by LC/F1 and LC/F6 merely by about 10%. Conversion of Gln-33 to alanine caused a reduction in cleavage by LC/F1 of less than 10% and of about 22% by LC/F6. The effect evoked by Gln-33 to Ala is probably, in part, also due to the lost interaction with Tyr-316 and/or Glu-310 (Figure S2; [35,36]). Another possibly interacting amino acid, Ser-167, differs in LC/F6. As this residue was proposed to form main-chain–main-chain interactions [35], its replacement with cysteine is unlikely to cause effects on binding. The involvement in VAMP-2 binding can be ruled out for a further 20 of the 25 differing residues as they are located remotely from the VAMP-2 binding cleft. However, LC/F1 Lys-146 and its counterpart Glu-146 in LC/F6 possibly interact differently with VAMP-2 Gln-38, whose mutation to alanine exerts a stronger effect on the activity of LC/F6 (Figure 3 and Figure S3). Yet, this difference argues for a tighter interaction of Gln-38 with LC/F6, and thus, cannot explain the higher K_M_ of LC/F6. Secondly, the replacement of LC/F1 Gly-177 with aspartic acid in LC/F6 might cause VAMP-2 repulsion through the vicinity to Val-48, thereby possibly explaining the higher K_M_ of LC/F6. According to this hypothesis, the VAMP-2 mutation Val-48 to Arg should lead to a clash with Asp-177 of LC/F6; however, this mutation compromises the activity of LC/F1 and LC/F6 to a similar low extent (Figure 3). Though few amino acids at the substrate–LC interface differ between LC/F1 and LC/F6, subtle discrepancies in VAMP-2 positioning must exist in order to explain the lower K_M_ and higher k_cat_ of LC/F1. Superposition of structural models for LC/F6, LC/F7, and LC/F9 on the structure of the LC/F1-VAMP-2 inhibitor peptide allowed prediction of differences in intermolecular electrostatic interactions (Figure S2). This analysis suggests LC/F1 Lys-29 and Arg-263 to be involved in side-chain-mediated interactions, whereas this is not predicted for LC/F6. Conversely, LC/F6 Glu-110, Glu-315, and Tyr-168 might form hydrogen bonds (H-bonds) to VAMP-2, whereas the structural model did not indicate corresponding bonds for LC-F1. Protease activity studies for mutants of these positions are required for final conclusions.

### 3.2. Comparison of LC/F1 and LC/F9 Substrate Interactions

Seventy-six of the 438 amino acids of LC/F9 differ versus LC/F1. In LC/F1, seven of those amino acids were proposed to be involved in ionic or H-bond interactions with VAMP-2 [35]. Based on the assumption that the overall VAMP-2 binding mode of LC/F1 and LC/F9 is the same, it may be concluded that the changes of Ser-167 to cysteine and Glu-310 to glycine in LC/F9 should not affect substrate interaction, as both are mediated by backbone atoms. Secondly, H-bond formation of LC/F1 Thr-132 with VAMP-2 Glu-41 was not predicted by the Discovery Studio Visualizer software. Therefore, its replacement with asparagine in LC/F9 is likely not affecting VAMP-2 binding. In contrast, replacement of Arg-133 with valine, of Glu-164 with lysine, and of Arg-171 with alanine in LC/F9 should significantly impair binding to VAMP-2 as these residues are proposed to form strong side-chain-mediated electrostatic interactions, thereby explaining different K_M_ values (Table 2; Figure S2). The change of Arg-240 (presumable NH1 interaction with OD1 and OD2 of Asp-57) of LC/F1 to lysine in LC/F9 probably leads to loss of the interaction with Asp-57, which may reduce k_cat_, since Asp-57 represents the important S_2_ position. The role of Arg-133 and Glu-164 in VAMP-2 interaction is less clear. Mutation of each reduces the catalytic activity of LC/F1 [35], whereas cleavage analysis of their proposed major VAMP-2 bonding partners, Asp-44 (presumably OD1 to Arg-133 NH2) and Arg-56 (presumably NH1 to Glu-164 OE1), does not lead to significantly reduced cleavage [29,36]. However, the Arg-133 NH1 interaction with the carbonyl oxygen of Val-43 may explain the existing data, and its absence due to replacement with valine in LC/F9 may add to the higher K_M_ value of LC/F9. A negative effect on the affinity of LC/F9 is also probable for the substitution of Glu-164 with lysine, most likely through an unfavorable space requirement of the longer lysine side chain or its charge, rather than through a lack of electrostatic interaction between LC/F1 Glu-164 and VAMP-2 Arg-56, because Arg-56 to Ala showed minimal effect on cleavage [29,36].

The pattern of hydrophobic interactions with VAMP-2 also differs between LC/F1 and LC/F9. Five of the 13 LC residues which were suggested to form hydrophobic interactions are replaced in LC/F9. Together, these changes are unlikely to be crucial for the different catalytic properties of the two LCs. Substitution of LC/F1 Leu-173 and Tyr-168 with serine, and Tyr-113 with lysine, causes an increase in K_M_ and a reduction in k_cat_ [36]. However, mutation of the suggested interaction partners of the former two, Ile-45 and Leu-54, respectively, bears similarly on hydrolysis by both LCs [29,36]. These data are in agreement with the prediction of electrostatic interactions of LC/F9 Ser-168 and Ser-173 with VAMP-2 (Figure S2, lowest panel; Figure S3), which compensate for the lost hydrophobic interactions. Similarly, a possible electrostatic interaction of LC/F9 Lys-113 with the backbone of VAMP-2 Asn-25 may at least compensate for the lost hydrophobic interaction with Leu-26. The exchanges Val-179 to Phe and Tyr-319 to Ile presumably do still allow hydrophobic interactions with their VAMP-2 binding partners, Val-53 and Leu-26, respectively.

The formation of novel interactions based on other replacements in LC/F9 cannot be excluded, and might be the reason why the mutation of several additional VAMP-2 residues, in particular Gln-33, Asp-40, Val-42, Asp-44, Val-48, Asp-51, Arg-56, and Gln-58 cause reduced cleavability by LC/F9 (Figure 3 and Figure S3). This may, in part, be due to an overall reduced affinity of LC/F9, so that minor disturbances lead to measurable effects. It may also indicate that positioning of VAMP-2 differs at some regions along the binding channel. More detailed analysis shall be the subject of future investigations.

### 3.3. Comparison of LC/F1 and LC/F7 Substrate Interactions

Among the VAMP-2 Q^58^–K^59^ hydrolyzing BoNT/F LCs, subtype 7 is the least related one. Strain CNM1212/11 (differs by Arg-368 to Lys from Oregon-CDC 59837) exhibits only 59.7–64.0% amino-acid sequence identity to the LC of F1, F2 F3, F4, F6, and F9 (Table S1). However, no clashes with VAMP-2 were observed upon superposition with coordinates for the co-crystal structure of VAMP-2-LC/F1 [35]. Which amino-acid changes can explain the lower affinity of LC/F7 (this study; [34])? Provided the substrate binding modes were the same, eight of the 13 amino acids of LC/F1 proposed to form electrostatic interactions with VAMP-2 differ in LC/F7 (Figure S2). LC/F1 Lys-29 being replaced by aspartic acid was suggested to interact with VAMP-2 via its side chain; however, later on, its involvement was experimentally disproved [35]. Substitution of LC/F1 Ser-167 with threonine and of LC/F1 Glu-310 with alanine is also unlikely to result in different interactions as those are formed via backbone atoms. Also, replacement of LC/F1 Asn-138 with glutamic acid should not significantly affect K_M_, as both residues may form similar interactions with VAMP-2 Gln-34 backbone residues. It is difficult to envisage that substitutions Arg-240 and Arg-263 of LC/F1 with lysine do explain the higher affinity of LC/F1. Both residues form electrostatic interactions with Asp-57 (P_2_ position; Figure S3), whose mutation dramatically reduces VAMP-2 cleavage by LC/F1 and LC/F7; however, mutation of Arg-240 and of its counterpart Lys-232 show comparable effects only on k_cat_ [34,36]. Information on the counterpart of LC/F1 Arg-263 (LC/F7 Lys-255) is missing. It might be that Lys-255 interacts less efficiently with VAMP-2, and thus, contributes to the higher K_M_ of LC/F7 (Table 2). This is supported by the lack of predicted electrostatic contacts for both lysine residues of LC/F7 (Figure S2). A second candidate that might cause higher affinity of LC/F1 is Tyr-244. Its substitution with asparagine leads to loss of interaction with the backbone carbonyl of Arg-56. However, the corresponding Asn-236 of LC/F7 is apparently involved in VAMP interaction, as Asn-236 to Ala causes a 7.5-fold reduction in activity [34]. According to structure analyses, different conformations of the conserved residues Arg-133 and Glu-164 might be responsible for the lower affinity of LC/F7. On the other hand, replacement of LC/F1 Ser-147 by aspartic acid appears to allow tighter interaction of LC/F7 with Gln-34 since the effect of the VAMP-2 Gln-34 to Ala mutation affects proteolysis by LC/F7 much stronger compared to LC/F1 (Figure 3). Similarly, LC/F7 Thr-146 in place of lysine may lead to novel electrostatic interactions with Gln-38 (Figure S2). This is supported by a strong effect of the Gln-38 to Ala mutation on cleavage by LC/F7 and a lesser effect on LC/F1 (Figure 3). Also, in favor of higher affinity of LC/F7 are predicted electrostatic interactions for Glu-110, Asn-131, Asn-176, and Tyr-307 (Figures S2 and S3).

In addition, variation in hydrophobic side-chain interactions may also account for the different K_M_ values. Data from VAMP-2 scanning mutagenesis showed mutation of Leu-32 and of Met-46 (Figure 3 and Figure S3) caused a much stronger effect on cleavage by LC/F7, whereas mutation of Leu-54 led to a stronger effect on cleavage by LC/F1. It is difficult to reconcile these differences on the basis of amino-acid changes between the two subtypes; e.g., mutation of Trp-44 in LC/F1 and LC/F7, which is supposed to stay in interaction with Leu-32, causes similar effects on the activity of both subtypes [34,36]. The change in the second residue proposed to be part of the binding pocket for Leu-32 from isoleucine (LC/F1) to methionine (LC/F7) is unlikely to explain the dramatically different effect of mutation Leu-32 to Ala. Secondly, based on the LC/F1-VAMP-2 co-crystal coordinates, VAMP-2 Met-46 is not involved in a close interaction. Though the amino acid located in its neighborhood, Gly-177, is replaced with an asparagine residue, there is no explanation for the strong effect of Met-46 to Ala on LC/F7 versus the very minor effect on LC/F1 (Figure 3; [29,36]). Although both LCs hydrolyze the same peptide bond in VAMP-2, these considerations together argue for significant local differences of VAMP-2 binding.

## 4. Conclusions

We identified a novel BoNT/F subtype, F9, and characterized the catalytic properties of its enzymatic domain in comparison to LC/F6 and F7 and the prototype F1. The study revealed that each subtype exhibits unique catalytic properties and substrate requirements, a fact that should be considered in inhibitor design. Our findings also imply that subtypes inherently differ with respect to clinically relevant substrate isoform specificity and intraneuronal duration of action, and that they are, therefore, of interest for in-depth biochemical investigations.

## 5. Materials and Methods 

### 5.1. Isolation of the Strain H078-01 Harboring BoNT/F9

Honey, whose consumption has been generally linked to infant botulism [37], was routinely screened for the presence of BoNT-producing clostridia. Honey (150 g) was dissolved in 1500 mL of water containing 0.1% Tween 80 for 30 min at 65 °C. Spores were collected via centrifugation for 30 min with 9000–12,000× *g* at 4 °C, washed with 20–30 mL of 0.1% Tween 80, and centrifuged again. The resulting sediment was re-suspended in 10 mL of Trypticase-Peptone-Glucose-Yeast extract-medium and cultivated anaerobically (10% H_2_, 10% CO_2_, 80% N_2_) at 32 °C in an anaerobic workstation (Don Whitley Scientific, Shipley, UK). Isolates were screened for the presence of *bont* genes by quantitative PCR [38]. An isolate from a commercial honey (mixture of EU and non-EU origin) was found positive for *bont/F*. The BoNT/F gene sequence was obtained as previously described [12], before being compared to other BoNT/F subtypes [30], and it is available under Genbank accession no. KX671959.1. The species was identified based on its 16S rDNA sequence as previously described [30].

### 5.2. Sequence Determination of C. baratii CNM1212/11

Strain CNM1212/11 was isolated from the first food-borne botulism outbreak in Europe which occurred in 2011 in Barcelona, Spain [27]. Genomic DNA was prepared from an overnight culture in TPGY using the Qiagen DNeasy Blood and Tissue Kit (Qiagen, Hilden, Germany) according to the protocol for Gram-positive bacteria. Genomic DNA was subjected to whole-genome sequencing on an Ion PGM (ThermoFisher Scientific, Waltham, MA, USA). Reads were assembled on *C. baratii* ATCC43256 (accession no. Y12091) [39] using Geneious (Biomatters Ltd., Auckland, New Zealand) [12]. The sequence of the neurotoxin gene is available under Genbank accession no. KX671958, and the derived amino-acid sequence was found to be >99.4% identical to other *C. baratii* BoNT/F7 amino-acid sequences.

### 5.3. BoNT Sequence Analysis

Amino-acid and nucleotide sequence comparisons of full-length BoNTs were performed using the software, Geneious 10.0.5, and the MAFFT algorithm [40]. The selection of subtype prototypes is based on Reference [6], with the following GenBank accession numbers used: F1, ABS41202 and GU213203; F2, CAA73972 and Y13631; F3, ADA79575 and GU213227; F4, GU213221; F5, GU213212; F6, AAA23263 and CP006903; F7, KX671958; F8, AUZC01000000; and H (FA, HA mosaic), KGOO15617 and JSCF01000000. BoNT amino-acid sequences were aligned using the built-in alignment tool (Blosum 62 matrix) and an Unweighted Pair Group Method with Arithmetic mean (UPGMA) consensus dendrogram was calculated from it (100 bootstrap replications, no outgroup). For comparison of BoNT subdomains, the region of the LC, H_N_, and H_C_ domains were extracted from the initial alignment of the full-length BoNT and pairwise identities calculated.

### 5.4. Plasmid Constructions

The cytosolic portion (amino acids 1–97) encoding segment of the wild-type rat VAMP-2 gene was inserted into the plasmid pET15b (Merck Biosciences GmbH, Schwalbach Ts., Germany). Point mutations in pET15b-VAMP-2 were introduced by oligonucleotide primer-based PCR mutagenesis using Pwo-Polymerase (Peqlab Biotechnologie GmbH, Erlangen, Germany) or the GeneTailor site-directed mutagenesis system (Invitrogen Corporation, Carlsbad, CA, USA). Nucleotide sequences of all mutants were verified by DNA sequencing.

*Escherichia coli* codon-optimized DNA segments encoding the LCs of BoNT/F1 (strain NCTC 10281 Langeland; accession: X81714) and of BoNT/F6 (strain 202F; accession: M92906 [41]), as well as native DNA segments of BoNT/F7 (strain *C. baratii* CNM1212/11; accession: KX671958), and of BoNT/F9 (strain H078-01; accession: KX671959.1) were cloned in the plasmid pH6F3H_C_ES [42], which allows for the production of LCs carrying an N-terminal His6-tag followed by a triple Flag-tag, and a C-terminal Strep-tag (IBA GmbH) using the *Bam*HI and *Sma*I sites.

### 5.5. Purification of Recombinant Proteins

The *E. coli* strain M15pREP4 (Qiagen GmbH, Hilden, Germany) was used for the production of LCs, whereas the strain BL21-DE3 (Stratagene Europe, Ebsdorfergrund, Germany) was used for VAMP-2. *E. coli* cultures were induced for 15 h at 21 °C, and proteins were purified on Ni^2+^–nitrilotriacetic acid–agarose beads (Qiagen GmbH, Hilden, Germany) according to the manufacturer’s instructions. Fractions containing the desired proteins were dialyzed against toxin assay buffer (150 mM potassium glutamate, 10 mM HEPES-KOH, pH 7.2), frozen in liquid nitrogen, and kept at −70 °C. Protein concentrations were determined following SDS-PAGE analysis and Coomassie blue staining by means of the LAS-3000 imaging system and the AIDA 3.51 program (both Fuji Photo Film, Co., Ltd., Tokyo, Japan), using various known concentrations of bovine serum albumin as standards.

### 5.6. In Vitro Transcription and Translation

VAMP-2 (amino acids 1–97) and its mutants were generated by in vitro transcription/translation using pET15b-VAMP-2, T7 polymerase, the TNT reticulocyte lysate system (Promega, Mannheim, Germany), and [^35^S] methionine (370 KBq/µL, >37 TBq/mmol, Hartmann Analytic, Braunschweig, Germany) according to the manufacturer’s instructions.

### 5.7. Endopep-MS-Assay

The Endopep-MS reaction was performed as described previously [11,43] with few modifications. Recombinant BoNT light chains (LC) were diluted in water to a final concentration of 25 µg/mL. Then, 2 µL of each toxin dilution was spiked in 16 µL of reaction buffer containing 50 mM HEPES (pH 7.3), 20 µM ZnCl_2_, and 25 mM dithiothreitol. Finally, 2 µL of peptide substrate T^27^SNRRLQQTQAQVDEVVDIMRVNVDKVLERDQ^58^K^59^LSELDDRADAL^70^ [11] of 5109.6 Da or full-length recombinant VAMP-2 substrate (amino acids 1–89) of 13816.7 Da was added to achieve a final concentration of 50 pmol/µL for the peptide reaction or 200 ng/µL (14.5 pmol/µL) for VAMP-2. The peptide substrate was synthesized by peptides & elephants (Potsdam, Germany) and VAMP-2 was purchased from ProSpec (East Brunswick, NJ, USA). The reaction solution was incubated at 37 °C for 1 h using a thermocycler. Subsequently, 2 µL of each reaction supernatant was mixed with 18 μL of MALDI-matrix 5 mg/mL α-cyano-4-hydroxycinnamic acid (Fluka, Buchs, Switzerland) dissolved in 50% acetonitrile (Carl Roth, Karlsruhe, Germany), 0.1% trifluoroacetic acid, and 1 mM ammonium citrate (both Sigma-Aldrich, Seelze, Germany). Then, 1 µL of this mixture was spotted on an MTP 384 polished steel target plate (Bruker Daltonics, Bremen, Germany). Mass spectra of each spot were acquired over the mass range *m/z* 1100 to 5500 in MS positive ion reflector mode (for peptide analysis) or *m/z* 2000 to 15,000 in MS positive linear mode (for protein analysis) on an autoflex speed matrix-assisted laser desorption/ionization time-of-flight (MALDI-TOF) mass spectrometer (Bruker Daltonics, Bremen, Germany) equipped with a smartbeam laser. For matrix suppression, deflection was set to *m/z* 1000 for peptide analysis and *m/z* 2000 for protein analysis. External mass calibration was performed with peptide calibration standard II or protein calibration I (both Bruker Daltonics, Bremen, Germany). Each spectrum represents an average of 3000 laser shots. Spectra were processed using the flexAnalysis 3.4 software (Bruker Daltonics, Bremen, Germany).

### 5.8. Endoprotease Assays

Cleavage assays contained *E. coli* expressed, purified VAMP-2 (50 µM) or 1 µL of transcription/translation mixture of [^35^S]-methionine-labeled VAMP-1 or VAMP-2 or VAMP-2 mutant and purified LC. Although VAMP-1 is the physiological target of BoNT, we used VAMP-2 as a surrogate substrate for mutant cleavage assays because the available structural information of the LC/F–substrate interaction is based on VAMP-2 [35], and it is cleaved with similar efficiency [44]. Proteins were incubated for 60 min at 37 °C in toxin assay buffer in a total volume of 10 µL. Reactions were stopped by the addition of an equal volume of double-concentrated sample buffer (120 mM Tris-HCl, pH 6.75, 10% (*v/v*) β-mercaptoethanol, 4% (*w/v*) SDS, 20% (*w/v*) glycerol, and 0.014% (*w/v*) bromophenol blue). Samples were incubated at 37 °C for 30 min, and then subjected to SDS-PAGE using 15% tris/tricine gels (acrylamide/bis-acrylamide in a 73.5:1 ratio). Subsequently, recombinant VAMP-2 was stained with Coomassie blue, whereas [^35^S]-labeled VAMPs were visualized after gel drying employing a FLA-9000 phosphorimager (Fuji Photo Film, Co., Ltd., Tokyo, Japan). The quantification of radiolabeled proteins and their cleavage products was done with the Multigauge 3.2 software (Fuji Photo Film, Co., Ltd., Tokyo, Japan).

For the determination of the enzyme kinetic parameters of LC/F subtypes, the substrate concentration was varied between 3 and 130 µM. Each of the various substrate concentrations was endowed by the addition of 1 µL of corresponding radiolabeled VAMP-2 generated by in vitro transcription/translation. Incubation was done in a final volume of 25 µL of toxin assay buffer. After 2 and 4 min of incubation at 37 °C, aliquots of 10 µL were taken, and the enzymatic reaction was stopped by mixing with 10 µL of pre-chilled double-concentrated SDS-PAGE sample buffer. The percentage of hydrolyzed VAMP-2 was determined from the turnover of the radiolabeled substrate, and was used to calculate the initial velocity of substrate hydrolysis. The K_M_ and V_max_ values were calculated by non-linear regression using the GraphPad Prism 4.03 program (GraphPad Software Inc, San Diego, CA, USA).

### 5.9. Protein Structure Analyses

Homology modeling of the LC/F subtype structures was done using the SWISS-MODEL server [45]. Structure analyses and structure superposition were done using the Discovery Studio Visualizer 2.5 software (Accelrys, Cambridge, UK) employing the Protein Data Bank (PDB) identifier 3FIE (LC/F1-inhibitor1 [35]).

## Figures and Tables

**Table 1:** Amino-acid identity comparisons of botulinum neurotoxin (BoNT)/F9 and other F subtypes.

F9 H078-01 vs.		% Pairwise Identity			
Subtype	Strain	LC	H_N_	H_C_	Holotoxin
F1	Langeland	82.5	85.6	84.2	84.1
F2	CDC3281	85.4	93.5	90.3	89.7
F3	VPI4257	86.6	95.9	92.4	91.6
F4	CDC54089	82.2	86.4	83.0	83.8
F5	CDC54075	46.4	83.3	92.2	73.5
F6	202F	80.6	92.1	88.2	86.9
F7	CNM1212/11	60.4	74.7	73.1	69.0
F8	357	82.0	85.3	83.9	83.7
H (FA, HA)	CFSAN024410	47.5	58.8	46.5	50.8

Identities are shown for pairwise comparisons of BoNT/F9 with BoNT/F1 to F8 subdivided into light chain (LC), N-terminal domain (H_N_) and C-terminal domain (H_C_) of the heavy chain, and to the holotoxin. The sequences used for comparison were chosen according to prototype strains given in Peck et al. [6]. GenBank accession numbers used are: F1, ABS41202 [8]; F2, CAA73972 [8]; F3, ADA79575 [8]; F4, GU213221 [8]; F5, GU213212 [8]; F6, AAA23263 [26]; F7, KX671958 [27]; F8, AUZC01000000 [7]; and H (FA, HA mosaic; JSCF01000000 [28]).

**Table 2:** Catalytic properties of various BoNT/F LC subtypes.

LC	K_M_^1^ (µM)	SD	k_cat_^1^ (1/min)	SD	k_cat_/K_M_ (1/µM·min)
F1 ^2^	28.7	4.9	1395	212	48.6
F6	70.1	5.2	442	3.3	6.3
F7	75.3	6.5	2100	282	27.9
F9	44.1	9.4	746	96	16.9

^1^ Values represent means of three to four experiments. ^2^ Data taken for comparison from Reference [29].

## Supplementary Materials

The following are available online at http://www.mdpi.com/2072-6651/10/8/311/s1, Table S1: Nucleotide and amino-acid identity comparisons of BoNT/F LC subtypes, Figure S1: Cleavage of VAMP-1 by LC/F1, LC/F6, LC/F7, and LC/F9, Figure S2: Proposed H-bond interactions of LC/F6, LC/F7, and LC/F9 with VAMP-2 in comparison to LC/F1, Figure S3: Electrostatic surfaces of LC/F1 and of structural models for LCF/6, LC/F7, and LC/F9 overlaid with the VAMP-2 structure as found in the LC/F1-VAMP-2 inhibitor peptide structure.

## References

[1] Brunt J., Carter A.T., Stringer S.C., Peck M.W. (2018). Identification of a novel botulinum neurotoxin gene cluster in *Enterococcus*. FEBS Lett..

[2] Zhang S., Lebreton F., Mansfield M.J., Miyashita S.I., Zhang J., Schwartzman J.A., Tao L., Masuyer G., Martinez-Carranza M., Stenmark P. (2018). Identification of a botulinum neurotoxin-like toxin in a commensal strain of *Enterococcus faecium*. Cell Host Microbe.

[3] Zhang S., Masuyer G., Zhang J., Shen Y., Lundin D., Henriksson L., Miyashita S.I., Martinez-Carranza M., Dong M., Stenmark P. (2017). Identification and characterization of a novel botulinum neurotoxin. Nat. Commun..

[4] Hill K.K., Smith T.J. (2013). Genetic diversity within *Clostridium botulinum* serotypes, botulinum neurotoxin gene clusters and toxin subtypes. Curr. Top. Microbiol. Immun..

[5] Rossetto O., Pirazzini M., Montecucco C. (2014). Botulinum neurotoxins: Genetic, structural and mechanistic insights. Nat. Rev..

[6] Peck M.W., Smith T.J., Anniballi F., Austin J.W., Bano L., Bradshaw M., Cuervo P., Cheng L.W., Derman Y., Dorner B.G. (2017). Historical perspectives and guidelines for botulinum neurotoxin subtype nomenclature. Toxins.

[7] Giordani F., Fillo S., Anselmo A., Palozzi A.M., Fortunato A., Gentile B., Tehran D.A., Ciammaruconi A., Spagnolo F., Pittiglio V. (2015). Genomic characterization of Italian *Clostridium botulinum* group I strains. Infect. Genet. Evol..

[8] Raphael B.H., Choudoir M.J., Luquez C., Fernandez R., Maslanka S.E. (2010). Sequence diversity of genes encoding botulinum neurotoxin type F. Appl. Environ. Microbiol..

[9] Smith T.J., Hill K.K., Raphael B.H. (2015). Historical and current perspectives on *Clostridium botulinum* diversity. Res. Microbiol..

[10] Henkel J.S., Jacobson M., Tepp W., Pier C., Johnson E.A., Barbieri J.T. (2009). Catalytic properties of botulinum neurotoxin subtypes A3 and A4. Biochemistry.

[11] Kalb S.R., Baudys J., Webb R.P., Wright P., Smith T.J., Smith L.A., Fernandez R., Raphael B.H., Maslanka S.E., Pirkle J.L. (2012). Discovery of a novel enzymatic cleavage site for botulinum neurotoxin F5. FEBS Lett..

[12] Kull S., Schulz K.M., Weisemann J., Kirchner S., Schreiber T., Bollenbach A., Dabrowski P.W., Nitsche A., Kalb S.R., Dorner M.B. (2015). Isolation and functional characterization of the novel *Clostridium botulinum* neurotoxin A8 subtype. PLoS ONE.

[13] Wang D., Krilich J., Pellett S., Baudys J., Tepp W.H., Barr J.R., Johnson E.A., Kalb S.R. (2013). Comparison of the catalytic properties of the botulinum neurotoxin subtypes A1 and A5. Biochim. Biophys. Acta.

[14] Whitemarsh R.C., Tepp W.H., Bradshaw M., Lin G., Pier C.L., Scherf J.M., Johnson E.A., Pellett S. (2013). Characterization of botulinum neurotoxin A subtypes 1 through 5 by investigation of activities in mice, in neuronal cell cultures, and in vitro. Infect. Immun..

[15] Kalb S.R., Baudys J., Egan C., Smith T.J., Smith L.A., Pirkle J.L., Barr J.R. (2011). Different substrate recognition requirements for cleavage of synaptobrevin-2 by *Clostridium baratii* and *Clostridium botulinum* type F neurotoxins. Appl. Environ. Microbiol..

[16] Kalb S.R., Baudys J., Smith T.J., Smith L.A., Barr J.R. (2014). Three enzymatically active neurotoxins of *Clostridium botulinum* strain Af84: BoNT/A2, /F4, and /F5. Anal. Chem..

[17] Kalb S.R., Smith T.J., Moura H., Hill K., Lou J.L., Geren I.N., Garcia-Rodriguez C., Marks J.D., Smith L.A., Pirkle J.L. (2008). The use of Endopep-MS to detect multiple subtypes of botulinum neurotoxins A, B, E, and F. Int. J. Mass Spectrom..

[18] Schiavo G., Shone C.C., Rossetto O., Alexander F.C., Montecucco C. (1993). Botulinum neurotoxin serotype F is a zinc endopeptidase specific for VAMP/synaptobrevin. J. Biol. Chem..

[19] Yamasaki S., Baumeister A., Binz T., Blasi J., Link E., Cornille F., Roques B., Fykse E.M., Sudhof T.C., Jahn R. (1994). Cleavage of members of the synaptobrevin/VAMP family by types D and F botulinal neurotoxins and tetanus toxin. J. Biol. Chem..

[20] Kalb S.R., Baudys J., Raphael B.H., Dykes J.K., Luquez C., Maslanka S.E., Barr J.R. (2015). Functional characterization of botulinum neurotoxin serotype H as a hybrid of known serotypes F and A (BoNT F/A). Anal. Chem..

[21] Bhattacharjee Y. (2011). Panel selects most dangerous select agents. Science.

[22] Gill D.M. (1982). Bacterial toxins: A table of lethal amounts. Microbiol. Rev..

[23] Chen S. (2012). Clinical uses of botulinum neurotoxins: Current indications, limitations and future developments. Toxins.

[24] Davletov B., Bajohrs M., Binz T. (2005). Beyond BOTOX: Advantages and limitations of individual botulinum neurotoxins. Trends Neurosci..

[25] Foster K., Chaddock J. (2010). Targeted secretion inhibitors-innovative protein therapeutics. Toxins.

[26] Smith T.J., Hill K.K., Xie G., Foley B.T., Williamson C.H., Foster J.T., Johnson S.L., Chertkov O., Teshima H., Gibbons H.S. (2015). Genomic sequences of six botulinum neurotoxin-producing strains representing three clostridial species illustrate the mobility and diversity of botulinum neurotoxin genes. Infect. Genet. Evol..

[27] Lafuente S., Nolla J., Valdezate S., Tortajada C., Vargas-Leguas H., Parron I., Saez-Nieto J.A., Portana S., Carrasco G., Moguel E. (2013). Two simultaneous botulism outbreaks in Barcelona: *Clostridium baratii* and *Clostridium botulinum*. Epidemiol. Infect..

[28] Gonzalez-Escalona N., Thirunavukkarasu N., Singh A., Toro M., Brown E.W., Zink D., Rummel A., Sharma S.K. (2014). Draft genome sequence of bivalent *Clostridium botulinum* strain IBCA10-7060, encoding botulinum neurotoxin B and a new FA mosaic type. Genome Announc..

[29] Sikorra S., Henke T., Galli T., Binz T. (2008). Substrate recognition mechanism of VAMP/synaptobrevin-cleaving clostridial neurotoxins. J. Biol. Chem..

[30] Hill K.K., Smith T.J., Helma C.H., Ticknor L.O., Foley B.T., Svensson R.T., Brown J.L., Johnson E.A., Smith L.A., Okinaka R.T. (2007). Genetic diversity among botulinum neurotoxin-producing clostridial strains. J. Bacteriol..

[31] Pellett S., Tepp W.H., Whitemarsh R.C., Bradshaw M., Johnson E.A. (2015). In vivo onset and duration of action varies for botulinum neurotoxin A subtypes 1–5. Toxicon.

[32] Torii Y., Kiyota N., Sugimoto N., Mori Y., Goto Y., Harakawa T., Nakahira S., Kaji R., Kozaki S., Ginnaga A. (2011). Comparison of effects of botulinum toxin subtype A1 and A2 using twitch tension assay and rat grip strength test. Toxicon.

[33] Guo J., Chan E.W., Chen S. (2016). Mechanism of substrate recognition by the novel botulinum neurotoxin subtype F5. Sci. Rep..

[34] Guo J., Chan E.W., Chen S. (2016). Comparative characterization of botulinum neurotoxin subtypes F1 and F7 featuring differential substrate recognition and cleavage mechanisms. Toxicon.

[35] Agarwal R., Schmidt J.J., Stafford R.G., Swaminathan S. (2009). Mode of VAMP substrate recognition and inhibition of Clostridium botulinum neurotoxin F. Nat. Struct. Mol. Biol..

[36] Chen S., Wan H.Y. (2011). Molecular mechanisms of substrate recognition and specificity of botulinum neurotoxin serotype F. Biochem. J..

[37] Arnon S.S., Midura T.F., Damus K., Thompson B., Wood R.M., Chin J. (1979). Honey and other environmental risk factors for infant botulism. J. Pediatr..

[38] Kirchner S., Kramer K.M., Schulze M., Pauly D., Jacob D., Gessler F., Nitsche A., Dorner B.G., Dorner M.B. (2010). Pentaplexed quantitative real-time PCR assay for the simultaneous detection and quantification of botulinum neurotoxin-producing clostridia in food and clinical samples. Appl. Environ. Microbiol..

[39] East A.K., Bhandari M., Hielm S., Collins M.D. (1998). Analysis of the botulinum neurotoxin type F gene clusters in proteolytic and nonproteolytic *Clostridium botulinum* and *Clostridium barati*. Curr. Microbiol..

[40] Katoh K., Misawa K., Kuma K., Miyata T. (2002). MAFFT: A novel method for rapid multiple sequence alignment based on fast Fourier transform. Nucleic Acids Res..

[41] East A.K., Richardson P.T., Allaway D., Collins M.D., Roberts T.A., Thompson D.E. (1992). Sequence of the gene encoding type F neurotoxin of *Clostridium botulinum*. FEMS Microbiol. Lett..

[42] Mahrhold S., Strotmeier J., Garcia-Rodriguez C., Lou J., Marks J.D., Rummel A., Binz T. (2013). Identification of the SV2 protein receptor-binding site of botulinum neurotoxin type E. Biochem. J..

[43] Hansbauer E.M., Skiba M., Endermann T., Weisemann J., Stern D., Dorner M.B., Finkenwirth F., Wolf J., Luginbuhl W., Messelhausser U. (2016). Detection, differentiation, and identification of botulinum neurotoxin serotypes C, CD, D, and DC by highly specific immunoassays and mass spectrometry. Analyst.

[44] Yamamoto H., Ida T., Tsutsuki H., Mori M., Matsumoto T., Kohda T., Mukamoto M., Goshima N., Kozaki S., Ihara H. (2012). Specificity of botulinum protease for human VAMP family proteins. Microbiol. Immun..

[45] Biasini M., Bienert S., Waterhouse A., Arnold K., Studer G., Schmidt T., Kiefer F., Gallo Cassarino T., Bertoni M., Bordoli L. (2014). SWISS-MODEL: Modelling protein tertiary and quaternary structure using evolutionary information. Nucleic Acids Res..

