# Supplementary material for: Botulinum Neurotoxin F Subtypes Cleaving the VAMP-2 Q58–K59 Peptide Bond Exhibit Unique Catalytic Properties and Substrate Specificities
Source: Toxins (Basel). 2018 Aug 1;10(8):311. doi: 10.3390/toxins10080311 (PMC6116196; doi:10.3390/toxins10080311)
Supplement: Supplementary file 1 [file toxins-10-00311-s001.zip › toxins-323155-SI.pdf]

Supplementary Materials: Botulinum Neurotoxin F Subtypes Cleaving the VAMP-2 Q^{58}-K^{59} Peptide Bond Exhibit Unique Catalytic Properties and Substrate Specificities

Stefan Sikorra, Martin Skiba, Martin B. Dorner, Jasmin Weisemann, Mirjam Weil , Sylvia Valdezate, Bazbek Davletov, Andreas Rummel, Brigitte G. Dorner and Thomas Binz

Table S1. Nucleotide and amino acid identity comparisons of BoNT/F LC subtypes.

Light Chain		Subtype									
Subtype	Strain	F1	F2	F3	F4	F5	F6	F7	F8	**F9**	H
F1	Langeland		82.0	82.9	96.4	47.0	94.1	63.3	97.7	**82.5**	49.1
F2	CDC3281	*91.3*		97.5	82.9	45.9	81.5	59.7	81.5	**85.4**	46.8
F3	VPI4257	*91.8*	*98.9*		83.8	45.9	82.7	60.8	82.5	**86.6**	47.0
F4	CDC54089	*98.3*	*91.9*	*92.3*		47.3	93.4	64.0	96.4	**82.2**	49.3
F5	CDC54075	*64.8*	*63.6*	*63.7*	*64.7*		47.3	46.5	46.4	**46.4**	79.7
F6	202F	*97.6*	*91.3*	*91.9*	*97.4*	*64.8*		63.3	94.1	**80.6**	48.0
F7	CNM1212/11	*75.6*	*73.1*	*73.5*	*75.9*	*62.5*	*76.1*		62.9	**60.4**	48.5
F8	357	*99.0*	*91.3*	*91.6*	*98.3*	*64.5*	*97.6*	*75.4*		**82.0**	49.3
**F9**	**H078-01**	***91.4***	***93.4***	***93.9***	***91.6***	***64.2***	***91.2***	***73.9***	***91.5***		**47.5**
H (FA, HA)	CFSAN024410	*63.8*	*63.2*	*63.0*	*63.9*	*86.2*	*63.5*	*61.7*	*63.7*	***63.2***	

Shown are the percentages of nucleic acid (*italic*, lower left gray triangle) and amino acid (upper right triangle) identities among LC/F subtypes. GenBank accession numbers used are: F1: ABS41202 and GU213203, F2: CAA73972 and Y13631, F3: ADA79575 and GU213227, F4: GU213221, F5: GU213212, F6: AAA23263 and CP006903, F7: KX671958, F8: AUZC01000000, F9KX671959.1 and H (FA, HA mosaic): KGOO15617 and JSCF01000000.

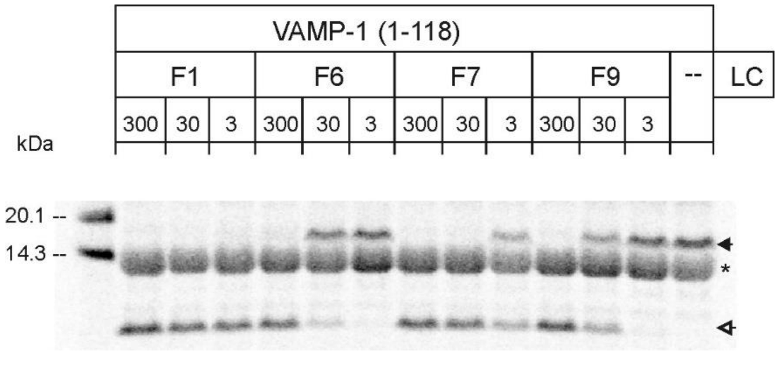

Figure S1. Cleavage of VAMP-1 by LC/F1, LC/F6, LC/F7, and LC/F9. Rat VAMP-1 (1–118) was generated by in vitro transcripton/translation and incubated with various LCF subtypes applied at 3, 30, and 300 nM final concentrations for 1 h at 37 °C in toxin assay buffer and subsequently subjected to SDS-PAGE. [^{35}S-Met]-labeled VAMP-1 and its cleavage fragments were visualized by phosphorimaging. The position of intact VAMP-1 is indicated by a filled arrowhead. The position of the C-terminal cleavage fragments is indicated by an open arrowhead. The N-terminal cleavage fragment does except for the initiation methionine not contain methionine and is therefore not detectable. The asterisk marks the position of hemoglobin non-specifically associated with [^{35}S-Met].

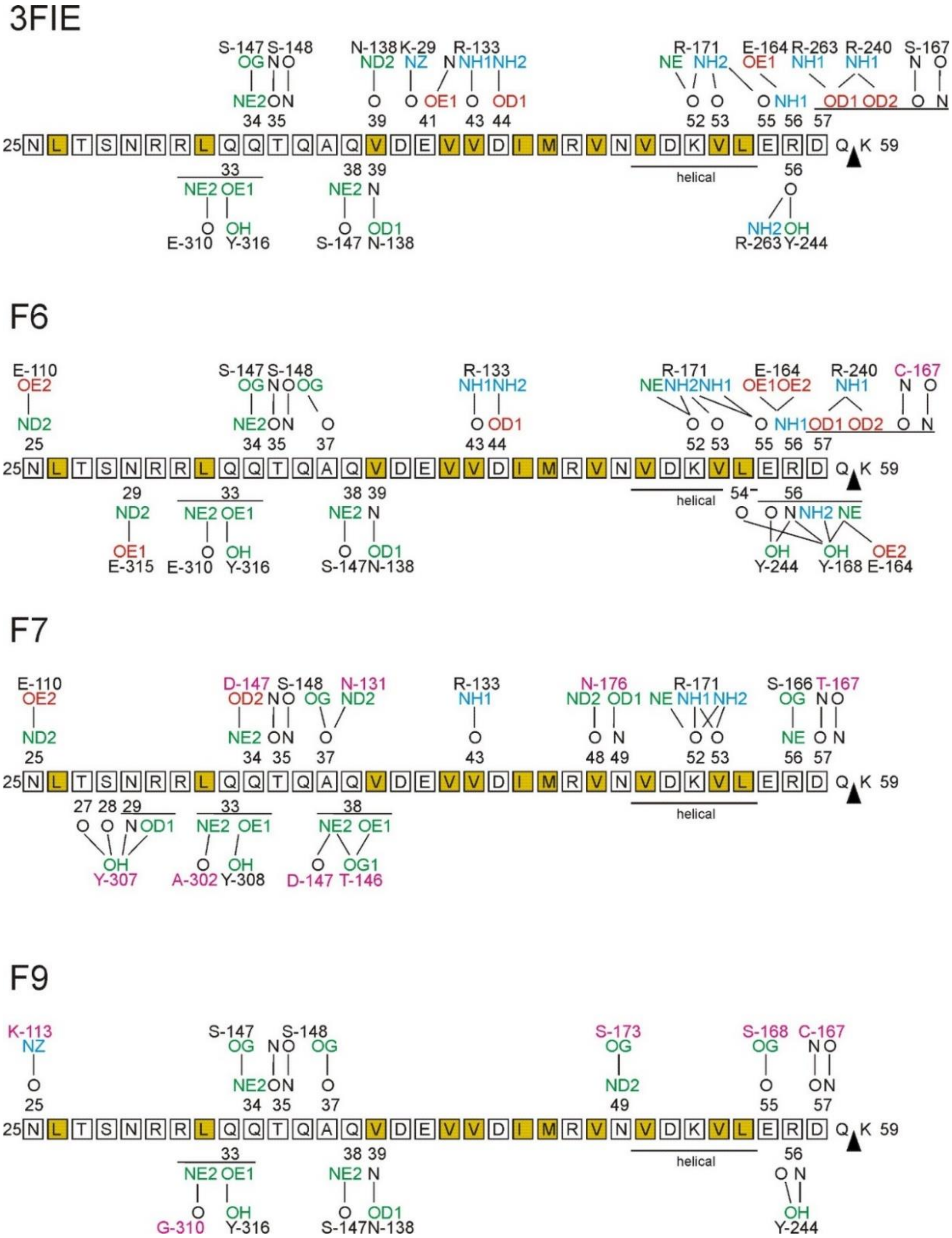

Figure S2. Proposed H-bond interactions of LC/F6, LC/F7, and LC/F9 with VAMP-2 in comparison to LC/F1. Structures for LC/F6, LC/F7, and LC/F9 were predicted applying the SWISS MODEL software [1] and then superimposed on the structure of the LC/F1/VAMP-2 inhibitor peptide 1 complex (PDB code: 3FIE) [2]) based on sequence alignments using the DS visualizer 2.5 software. Intermolecular H-bonds to VAMP-2 were calculated by using DS visualizer 2.5. VAMP-2 inhibitor peptide amino acids are boxed and shown in single letter code. Residues carrying hydrophobic side chains are highlighted yellow. Positions and atoms or functional groups are specified for those residues of VAMP-2 that form interactions with LC/F. LC/F amino acids proposed to H-bond to VAMP-2 are indicated including their participating atoms or functional groups. They are highlighted magenta for LC/F6, LC/F7, and LC/F9, if they differ from their counterpart in LC/F1. Uncharged side chain groups are presented in green, negatively charged groups in red, and positively charged groups in blue.

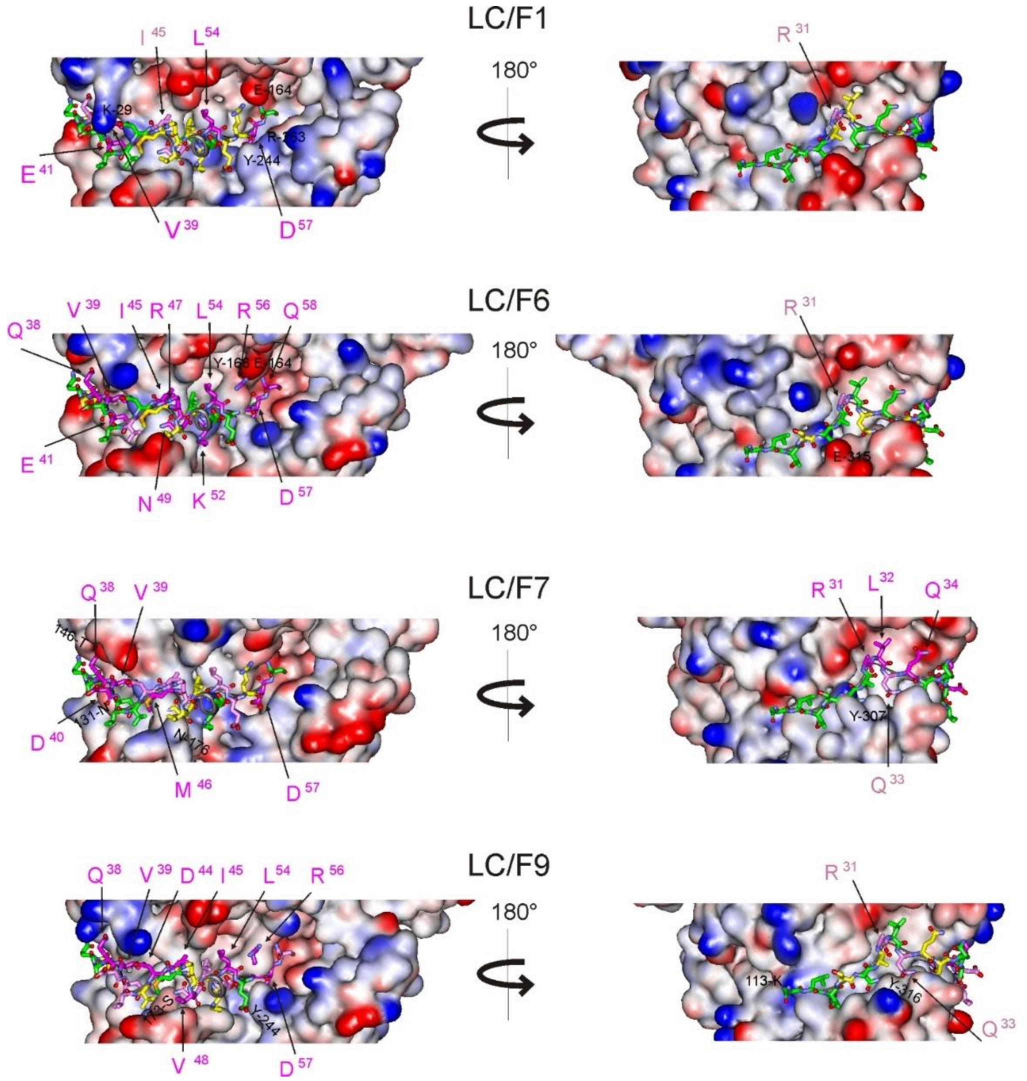

Figure S3. Electrostatic surfaces of LC/F1 and of structural models for LCF/6, LC/F7, and LC/F9 overlaid with the VAMP-2 structure as found in the LC/F1-VAMP-2 inhibitor peptide structure (PDB code: 3FIE; [2]). VAMP-2 residues are specified and colored in line with Figure 3 according to their importance for interaction with the respective LC/F. Where applicable LC residues proposed to interact with VAMP-2 are marked by their single letter code character on the respective surface position. Note that VAMP-2 Arg-30 and Arg-31 were disordered in the crystal structures complex and could thus not be modeled.

References

1. Biasini, M.; Bienert, S.; Waterhouse, A.; Arnold, K.; Studer, G.; Schmidt, T.; Kiefer, F.; Gallo Cassarino, T.; Bertoni, M.; et al. SWISS-MODEL: Modelling protein tertiary and quaternary structure using evolutionary information. *Nucleic Acids Res.* **2014**, *42*, W252–W258.
2. Agarwal, R.; Schmidt, J.J.; Stafford, R.G.; Swaminathan, S. Mode of VAMP substrate recognition and inhibition of *Clostridium botulinum* neurotoxin F. *Nat. Struct. Mol. Biol.* **2009**, *16*, 789–794.
